# Effects of Microcystin-LR on the Microstructure and Inflammation-Related Factors of Jejunum in Mice

**DOI:** 10.3390/toxins11090482

**Published:** 2019-08-21

**Authors:** Linghui Cao, Feiyu Huang, Isaac Yaw Massey, Cong Wen, Shuilin Zheng, Shuaishuai Xu, Fei Yang

**Affiliations:** Department of Occupational and Environmental Health, Xiangya School of Public Health, Central South University, 110 Xiangya Road, Changsha 410078, China

**Keywords:** MC-LR, mice, jejunum, inflammatory factor, chronic exposure

## Abstract

The increasing cyanobacterial blooms have recently been considered a severe environmental problem. Microcystin-leucine arginine (MC-LR) is one of the secondary products of cyanobacteria metabolism and most harmful cyanotoxins found in water bodies. Studies show MC-LR negatively affects various human organs when exposed to it. The phenotype of the jejunal chronic toxicity induced by MC-LR has not been well described. The aim of this paper was to investigate the effects of MC-LR on the jejunal microstructure and expression level of inflammatory-related factors in jejunum. Mice were treated with different doses (1, 30, 60, 90 and 120 μg/L) of MC-LR for six months. The microstructure and mRNA expression levels of inflammation-related factors in jejunum were analyzed. Results showed that the microstructure of the jejunum was destroyed and expression levels of inflammation-related factors interleukin (IL)-1β, interleukin (IL)-8, tumor necrosis factor alpha, transforming growth factor-β1 and interleukin (IL)-10 were altered at different MC-LR concentrations. To the best of our knowledge, this is the first study that mice were exposed to a high dose of MC-LR for six months. Our data demonstrated MC-LR had the potential to cause intestinal toxicity by destroying the microstructure of the jejunum and inducing an inflammatory response in mice, which provided new insight into understanding the prevention and diagnosis of the intestinal diseases caused by MC-LR.

## 1. Introduction

The rapid development of industries and agriculture has led to the increasing discharge of wastewater containing nitrogen and phosphorus into water bodies. These activities may result in eutrophication and development of cyanobacterial blooms [1,2]. The monocyclic heptapeptide microcystins (MCs) are the secondary products of cyanobacteria metabolism and most harmful cyanotoxin found in water bodies [3,4,5]. More than 200 isomers of MCs have been identified from cyanobacterial blooms, with MC-LR being the most widely distributed, abundant and toxic [1,6,7]. 

The health issues including diseases and deaths of animals and humans caused by MC-LR occur from time to time worldwide. Previous studies showed that animals and humans are mainly exposed to MC-LR through drinking polluted water, body contact, hemodialysis, consumption of contaminated food and algal dietary supplements [8,9,10]. When MC-LR is ingested, it first enters the intestine where most of these toxins are absorbed through the intestinal mucosal barrier (mucosal epithelial cells and mucosal lamina propria), and the absorbed MC-LR are transported through the bloodstream and distributed to the liver and other organs [11]. This may lead to the development of tumor and various acute liver diseases, nervous system damage, gastroenteritis problems and death [4,9,12,13,14,15]. 

The most serious human health issue associated with MC-LR exposure occurred in February 1996, where water used for hemodialysis at a hemodialysis center in Caruaru, Brazil was contaminated with MC-LR in the hospital's water supply leading to the death of over 50 patients following the development of acute liver failure, visual disturbances, nausea, and vomiting [16]. To combat MC-LR health-related problems, the World Health Organization stipulated that the concentration of MC-LR in drinking water should not exceed 1 μg/L [17]. 

The small intestine, consisting of duodenum, jejunum and ileum, is the most important part for food absorption and digestion [18]. While the duodenum mainly takes in minerals, the jejunum assimilates water-soluble vitamins, enzymes and proteins. The ileum absorbs fat, cholesterol and bile salts [19,20]. The digestive enzyme in the brush edge of intestinal villous epithelial cells also plays a key role in the final digestion stage, and its activity is closely related to the structural integrity of intestinal mucosa [21]. If inflammation or pathological changes occur in the small intestine, the digestion and absorption dysfunction of the small intestine may lead to the dysfunction of the human body [22]. It is reported that inflammation usually causes ulcerative colitis [23], Crohn's disease [24], and other related diseases [25]. The small intestine plays an irreplaceable role in the digestion, absorption and immune function of the human body, thus it’s important to investigate whether intestinal toxicity can be induced by MC-LR.

Immune response is often accompanied by inflammation [26,27], and inflammation is usually regulated by cytokines and chemokines [28,29]. Cytokines, which participate in immunomodulation, anti-tumor, hematopoiesis, inflammatory response, neuroendocrine effect and many other biological functions, are small peptides or glycoproteins produced by lymphocytes, monocytes, macrophages, stroma, endothelial cells and fibroblasts [30]. Chemokines are chemotactic cytokines originally identified as factors regulating immune cell migration to sites of inflammation and have an involvement in numerous physiological and pathological processes including immunity, inflammation, and neuroinflammation [31]. Cytokines such as tumor necrosis factor alpha (TNF-α) and interleukin (IL)-1β, and chemokines including interleukin (IL)-8, capable of driving inflammatory processes and accelerating additional inflammatory processes by inducing inflammatory molecules, were described as pro-inflammatory factors [31,32]. Cytokines such as interleukin (IL)-10 possessing the main function of reducing damage to an organism by inhibiting the production of pro-inflammatory factors were addressed as anti-inflammatory factors [33]. TNF-α, secreted by immune cells, is a key regulator of inflammation, and regulates leukocyte migration, proliferation, differentiation and apoptosis [34]. The IL-8, IL-1β and IL-10, members of the interleukin (IL) family, produced by lymphocytes, monocytes, or other non-monocytes, play an important part in immunomodulation, hematopoiesis and inflammation [35]. Cytokine transforming growth factor-β1 (TGF-β1) known to have many biological effects participates in inflammatory response, as well as inhibits the proliferation and differentiation of immune cells and the production of cytokines in the immune response [36]. Moreover, TGF-β1 has a dual role of pro- and anti-inflammatory effects however its dominant role is depended on the local microenvironment [37]. 

Though a number of population investigations have been conducted on the toxicity of MC-LR on humans [38,39], there are few studies regarding intestinal toxicity, pathological changes in jejunum and the expression level of inflammatory-related factors in jejunum under the induction of MC-LR. In this study, jejunal chronic toxicity induced by MC-LR was investigated. Here, a mice model was used to examine the effects of MC-LR on the jejunal microstructure and expression levels of inflammatory-related factors. The study aims to provide new evidence for understanding intestinal discomfort, inflammation and immunity caused by MC-LR.

## 2. Results

### 2.1. Appearance and Length of Small Intestine of Mice

No death or symptoms in MC-LR treated mice were recorded during the six-month experiment. Compared to the control group, no significant differences were observed in the MC-LR treated groups in terms of weight gain, diet, water consumption and activity, nor did the intestinal length/mice height ratio (Table 1).

### 2.2. Microstructure of Jejunum

**Figure 1:**
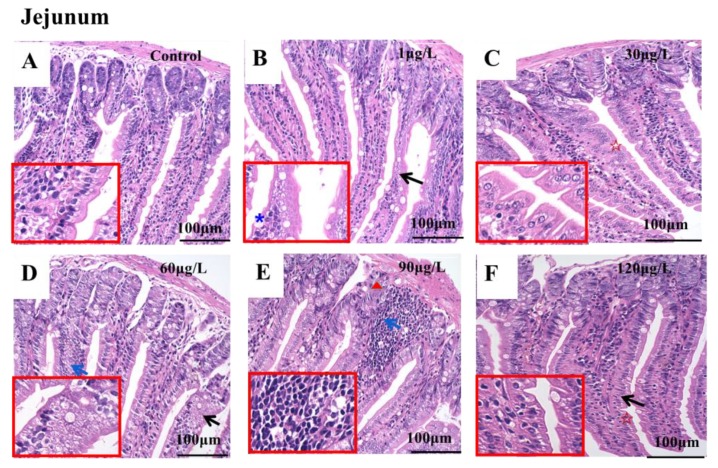
The effects of different doses of MC-LR on the histopathology of the jejunum in mice. **A**–**F** illustrates the microstructure of the jejunum in mice. Bar = 100 μm means original magnification ×400. Microstructure of the jejunum of mice in different groups (**A**, **B**, **C**, **D**, **E** and **F** represents the control group, 1 μg/L dose group, 30 μg/L dose group, 60 μg/L dose group, 90 μg/L dose group and 120 μg/L dose group, respectively). (**A**) The intestinal villi were well-structured and coherent, and the crypts were neatly arranged; **(B**) the jejunal microstructure showed that the goblet cells increased by 38%, and the arrangement of intestinal epithelial cells were disordered; (**C**) the intestinal villi were invaginated and serrated; (**D**) the arrangement of intestinal epithelial cells was disordered and lymphocyte infiltration was observed; (**E**) obvious lymphocyte infiltration and disorder crypts were observed; (**F**) the intestinal villi were found to be invaginated and serrated. The red five-pointed star indicates the intestinal villi were invaginated and serrated. The black arrow indicates an arrangement disorder of the intestinal epithelial cells, and the blue arrow shows the infiltration of lymphocytes. The blue asterisk indicates an increase in goblet cells. The red triangle indicates a disordered arrangement of crypts.

To evaluate the influence of MC-LR on the microstructure of the jejunum, hematoxylin and eosin staining (HE staining) were conducted as depicted in the method and photographed by using the Invitrogen EVOS FL Auto 2.0 Image System. The effects of different doses of MC-LR on the histopathology of mice jejunum are shown in Figure 1. In the control group, the intestinal villi were well-structured and coherent, and the crypts were neatly arranged. The surface of villi was covered with columnar cells, with a large number of cells arranged in the interphase with goblet cells, and the size of each cell was uniform. The mucosal lamina propria was intact and had no symptoms of separation and hyperemia (Figure 1A). In the 1 μg/L dose group, the jejunal microstructure showed that the goblet cells increased by 38%, and the arrangement of intestinal epithelial cells was disarrayed (Figure 1B). In the 30 μg/L dose group, the intestinal villi were invaginated and serrated (Figure 1C). In the 60 μg/L dose group, the arrangement of intestinal epithelial cells was also disarrayed and lymphocyte infiltration was observed (Figure 1D). In the 90 μg/L dose group, obvious lymphocyte infiltration and disorder crypts were observed (Figure 1E). In the 120 μg/L dose group, the intestinal villi were found to be invaginated and serrated (Figure 1F).

### 2.3. Effect of MC-LR on the Expression of Jejunum Inflammation-Related Factors in Mice

**Figure 2:**
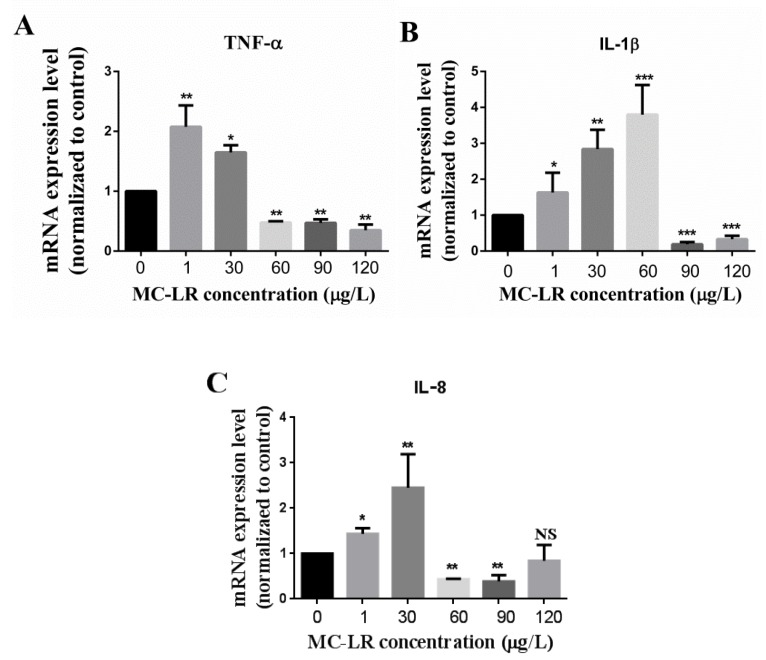
The effects of MC-LR on the pro-inflammatory factors mRNA expression levels of the jejunum in mice. (**A**) mRNA expression levels of pro-inflammatory factor TNF-α in 0, 1, 30, 60, 90 and 120 μg/L MC-LR treatment groups; (**B**) mRNA expression levels of pro-inflammatory factor IL-1β in 0, 1, 30, 60, 90 and 120 μg/L MC-LR treatment groups; (**C**) mRNA expression levels of pro-inflammatory factor IL-8 in 0, 1, 30, 60, 90 and 120 μg/L MC-LR treatment groups. * indicates *p* <0.05, ** indicates *p* <0.01, *** indicates *p* <0.001, NS means no significance.

**Figure 3:**
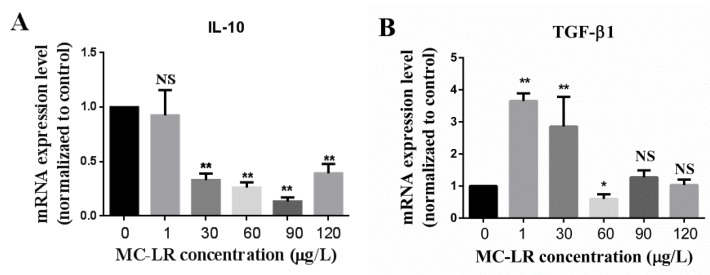
The effects of MC-LR on the anti-inflammatory factors mRNA expression levels of the jejunum in mice. (**A**) mRNA expression levels of anti-inflammatory factor IL-10 in 0, 1, 30, 60, 90 and 120 μg/L MC-LR treatment groups; (**B**) mRNA expression levels of anti-inflammatory factor TGF-β1 in 0, 1, 30, 60, 90 and 120 μg/L MC-LR treatment groups. *indicates *p* <0.05, ** indicates *p* <0.01, NS means no significance.

In order to determine whether MC-LR has an effect on the expression levels of inflammation-related factors in jejunum, the jejunum was isolated as described in the method. Total RNA was extracted and qRT-PCR was carried out as presented in the method. The changes in transcriptional levels of inflammatory-related factors including pro-inflammatory factors (IL-1β, IL-8, and TNF-α) and anti-inflammatory factors (IL-10 and TGF-β1) in the jejunum of mice are shown in Figure 2 and Figure 3, respectively. Data demonstrated that the pro-inflammatory factor TNF-α was up-regulated in the 1 μg/L and 30 μg/L dose groups, but down-regulated in the 60 μg/L, 90 μg/L and 120 μg/L dose groups (Figure 2A). IL-1β was up-regulated in the 1 μg/L, 30 μg/L and 60 μg/L dose groups, but down-regulated in the 90 μg/L and 120 μg/L dose groups (Figure 2B). IL-8 was also up-regulated in the 1μg/L and 30 μg/L dose groups, but down-regulated in the 60μg/L and 90 μg/L dose groups while no significant alteration was observed in the 120 μg/L dose group (Figure 2C). Although the anti-inflammatory factor IL-10 did not change significantly in the 1 μg/L dose group, it was notably down-regulated in the 30 μg/L, 60 μg/L, 90 μg/L and 120 μg/L dose groups (Figure 3A). In addition, the expression level of TGF-β1, also an anti-inflammatory factor was up-regulated in the 1 μg/L and 30 μg/L dose groups, but down-regulated in the 60 μg/L dose group while other differences were not significant (Figure 3B). 

## 3. Discussion

The eutrophication of water is currently on the increase and has become a worldwide serious water pollution problem. The outbreak of cyanobacterial blooms and the production of MC-LR do not only affect the ecological environment but also pose a great health threat to humans and animals [9]. In this study, the effect of MC-LR on jejunum was investigated by the method of chronic oral exposure. After six months of oral MC-LR exposure, the physical appearances of mice were examined, the microstructure of jejunum was observed by HE staining, and the mRNA expression levels of inflammatory-related factors were evaluated. In the six-month experiment, MC-LR-treated mice showed no signs of death or other abnormalities. There were no significant differences in weight gain, diet, water consumption and activity. In addition, no abnormal intestinal length/mice height ratio between the MC-LR-treated groups and control group was observed. These results were consistent with what Sedan et al. [40] reported in the toxicity study of mice exposed to a low-dose of MC-LR. In addition, a sub-acute study carried out by Su et al. [41] also demonstrated no alteration in body weight. However, Pan et al. [42] explored the toxicity of MC-LR on mice prostate at a concentration of 10 μg/L and 30 μg/L for 90 and 180 days, and reported a significant decrease in mice body weight treated with 30  μg/L MC-LR in comparison with the control group while the body weight of mice markedly increased after exposure to 10  μg/L MC-LR. In view of this, it is speculated that MC-LR might have an effect on the metabolic enzymatic activity in mice, while the latter was related to the survival conditions of the mice. 

The microstructure experimental results showed that at a low MC-LR concentration of 1 μg/L, which is the upper limit specified by the WHO in human’s drinking water, can induce pathological changes in jejunum (Figure 1B) [17]. Further varying degrees of alterations in other higher dose groups, including disordered intestinal epithelial cells (Figure 1B) and crypts (Figure 1B and Figure 1D), invaginated and serrated intestinal villi (Figure 1C and Figure 1F), and infiltrated lymphocytes (Figure 1E and Figure 1D) were apparent. In view of this, we may infer that MC-LR might destroy the intestinal integrity since Baumgart et al. demonstrated that the complete intestinal barrier includes four parts: Mechanical barrier, biological barrier, immune barrier and chemical barrier, while the intestinal epithelial cells and crypts are disorderly arranged, and the serration and invagination of intestinal villi belongs to the mechanical barrier of intestine [43]. Ren et al. [44] and Peterson et al. [45] indicated that the mechanical barrier of the intestine is not only key to protecting the intestinal tract against invasion of the intestinal mucosa by pathogens or harmful substances from the external environment, but also forms the structural basis for maintaining the selective permeability of the intestinal epithelium and its barrier function. Moreover, the mechanical barrier of the intestine is a regulator of immune homeostasis. Our findings were similar to those of Ito et al. [46,47] where villi density, surface erosion and lamina propria of the small intestine in mice with different alterations after oral administration of 500 μg MC-LR/kg were addressed. Evidence thus suggests that MC-LR can destroy the physical structure of the small intestine when exposed to it.

Studies suggest that MC-LR is capable of inducing modulatory potency on the immune system in vivo and in vitro. Sedan et al. [40] reported a decrease in intraepithelial lymphocytes in the mucous of the intestine when mice were orally exposed to 50 μg/kg and 100 μg/kg MC-LR for 48 h. Chen et al. [48] demonstrated an increase in mRNA levels of IFN-1, IL-1β, IL-8, TGF-β and TNF-α when zebrafish were exposed to 0 μg/L, 1 μg/L, 5 μg/L and 20 μg/L MC-LR for 30 days. Li et al. [49] revealed that MC-LR up-regulated the transcription of several genes essential for early lymphoid development and the production of heat shock proteins, potentially causing changes in the functioning of the immune system in zebrafish larvae. Rymuszka et al. [50] also reported an increase in the respiratory burst activity of phagocytic cells at the lowest toxin concentration but it was decreased at higher concentrations when leucocytes (lymphocytes and phagocytes) were treated with MC-LR at concentrations of 0.01 μg/mL, 0.1 μg/mL, 0.5 μg/mL and 1μg/mL. A decrease in the proliferation of B lymphocytes was detected in MC-LR-treated groups. Additionally, MC-LR induced necrosis to a higher degree than apoptosis in fish leucocytes [50]. Recently Xia et al. found that MC congener MC-RR can also regulate the immune system aside MC-LR by accumulating in the kidney and spleen, which are the major immune organ in fish, and cause further damage to these organs [51]. Cytokines and chemokines are well known to have an involvement in the immune response. For the process of the immune response, the inflammation response usually occurs first. IL-1β is one of the most sensitive and potent inflammatory factors reported [52]. When there is an inflammatory stimulus, the expression level of the IL-1β rapidly increases but decreases over time. This decrease may be due to the synthesis of the inhibition of the transcription and/or lessen of the half-life of the mRNA [53]. In this study, the expression level of IL-1β was up-regulated in the 1 μg/L, 30 μg/L, and 60 μg/L dose groups and down-regulated in the higher dose groups (Figure 2B). These might be attributed to the above reasons. TNF-α regulates inflammation, mucus secretion, cell survival and death in the small intestine [54]. The current data showed that the expression level of TNF-α was up-regulated in the 1μg/L and 30 μg/L dose groups, but down-regulated in the 60 μg/L, 90 μg/L and 120 μg/L dose groups. IL-8, involved in anti-infection, immune response regulation and anti-tumor, is a potent chemoattractant for neutrophils and contributes to inflammation [55]. In this study, IL-8 was also up-regulated in the 1μg/L and 30 μg/L dose groups, but down-regulated in the 60 μg/L and 90 μg/L dose groups while no significant alteration was observed in the 120 μg/L dose group. (Figure 2A and Figure 2C, respectively). These noted above indicated that MC-LR can promote the production of inflammatory factors, leading to the occurrence of the inflammatory response in mice at the lower concentrations. That is, under the stimulation of a low MC-LR concentration, the pro-inflammatory factors mRNA of TNF-α, IL-1β and IL-8 were synthesized and released. However, after a high-dose and long-term stress of MC-LR, the immune system of mice might be seriously damaged (as depicted in Figure 1) and inhibit the immune response. Pan et al. [42] and Rymuszka et al. [50] reported that a higher concentration of MC-LR could decrease the viability and increase the apoptosis or necrosis of cells. Further, Freire et al. [56], Savill et al. [57,58] and Voll et al. [59] demonstrated that apoptotic or necrotic cells can be engulfed and digested by phagocytes, thus avoiding the leakage of toxic substances or immunogenic substances in cells, so as to reduce body damage. Phagocytes that engulfed apoptotic and necrotic cells inhibit the immune response. For example, after phagocytosis of apoptotic granulocytes, macrophages secrete anti-inflammatory factors such as TGF-β, which inhibit the production and secretion of cytokines and chemokines MIP-2 (macrophage inflammatory protein 2), IL-1β and TNF-α [57,58,59]. Consequently, it is also possible that after a long-term higher dose of MC-LR induction, cells can no longer secrete inflammatory factors due to its apoptosis or necrosis. As a result, the expression levels of TNF-α, IL-1β and IL-8 were decreased in the higher dose groups. Nevertheless, the reasons why the expression levels of inflammation-related factors were altered are worth researching. Further work needs to detect the changes in the intestinal cell state (alive or dead), immune cell and immune response under higher concentrations of MC-LR.

The cytokine TGF-β1, was up-regulated in the 1 μg/L and 30 μg/L dose groups, but down-regulated in the 60 μg/L dose group while other groups differences were not significant (Figure 3B). Although TGF-β1 can be either an anti- or pro-inflammatory factor depending on the circumstances, it is also temporarily studied as an anti-inflammatory factor in this current study as most articles consider it as an anti-inflammatory factor [60,61,62] while fewer articles treat it as a pro-inflammatory factor [63]. Another anti-inflammatory factor IL-10 also possesses the potency of inhibiting the production of IL-1β, IL-8, and TNF-α, so as to reduce the inflammation of tissue. Our results also demonstrated that though the IL-10 mRNA expression level did not change significantly in the 1 μg/L dose group, it was remarkably down-regulated in higher dose groups (Figure 3A). It is therefore speculated that the anti-inflammatory effects of TGF-β1 and IL-10 may differ at different concentrations.

These results suggested that the up-regulation of pro-inflammatory factors IL-1β, IL-8 and TNF-α can aggravate the inflammatory reaction and microstructure damage of jejunum after oral exposure to MC-LR in mice. Anti-inflammatory factors, IL-10, plays a protective role by inhibiting many of the above factors. TGF-β1, reported as a pro- and anti-inflammatory factor depending on the local microenvironment [37], was considered as an anti-inflammatory factor in the current study due to substantial reports on its effect of anti-inflammation. Our data indicated that MC-LR could induce an inflammatory reaction, which is similar to what Su et al. [41] found in the toxicity study on the development of pre-existing colitis in mice. In short, the imbalance of pro- and anti-inflammatory factors resulted in the injury of the jejunal microstructure and the alterations of expression levels of inflammation factors, thereby affecting the immune response of the whole intestine.

## 4. Conclusions

To the best of our knowledge, this is the first study that mice were exposed to a high dose of MC-LR for six months. Findings demonstrated that a long-term chronic high-dose oral exposure to MC-LR has the potential to cause intestinal toxicity by destroying the microstructure of the jejunum and inducing an inflammatory reaction in mice. These results provide a new direction for future research and make contribution to the prevention, diagnosis and treatment of intestinal diseases caused by MC-LR even though the involvement of the mechanism of toxicity in the damage of the small intestine should be studied in depth.

## 5. Materials and Methods 

### 5.1. Mice and Reagents

C57BL/6J mice (age: 6–8 weeks; weight: About 20 g) were used in this study. The mice were procured from the Experimental Animal Centre of Central South University, China. Standard guidelines (humidity: 60% ± 5%; temperature: 24 °C± 3 °C) were used to store the animals for 12 h light and dark cycle. All experiments were approved by the Animal Care and Use Committee of the Central South University (identification code: XYGW-2018-41; date of approval: 10 November 2018). In this study, a total of 60 mice were randomly divided into the following six groups (n = 10): Five test groups, including MC-LR at the dose of 1, 30, 60, 90, and 120 μg/L in 0.012% (*v/v*) dimethyl sulfoxide (DMSO) for drinking water, respectively and one solvent (0.012% DMSO) control group. The mice in each group were exposed to drinking water containing MC-LR at the corresponding concentrations for six months. MC-LR was purchased from Taiwan Algal Science Inc. (Taiwan, China). DMSO (purity of ≥99.7%) was obtained from Sigma-Aldrich (St. Louis, MO, USA). 

### 5.2. Tissue Anatomy

After six months of MC-LR exposure, the mice were weighed and killed. Small intestinal samples were immediately isolated and measured. The blood stains were washed out with phosphate buffered solution (PBS), and then fixed with 4% paraformaldehyde (PFA).

### 5.3. Hematoxylin and Eosin Staining

Fixed intestinal tissues were embedded in the paraffin, continuous slice, and thickness of 4 μm. The intestinal section was dehydrated at a gradient alcohol routinely [64], xylene was used as a transparent agent, hematoxylin and eosin (HE) staining, dehydration and mounting. Observation and photography were done under the Invitrogen EVOS FL Auto 2.0 Image System (Thermo Fisher Scientific, Shanghai, China). Quantification of the histological analysis was conducted as described previously [65].

### 5.4. Quantitative RT-PCR

Total RNA was isolated from jejunal tissues (including the control and MC-LR-treated groups) using Trizol (Life Technologies, Shanghai, China), and reverse-transcribed into cDNA with Superscript III reverse transcriptase (Invitrogen, Shanghai, China). qRT-PCR was performed with a Roche Light Cycle 96 instrument with the SYBR (TaKaRa, Dalian, China) system and a thermal profile of 40 cycles at 95 °C for 10 s and 60 °C for 15 s [66]. The primer 3.0 online software was used to design primers involved in this paper. The suitability of primers was detected by means of the reverse transcription PCR (RT-PCR). cDNA was used as template and corresponding primers were used to perform PCR (the PCR program was the same as that of qRT-PCR), and PCR products were detected by gel electrophoresis. Electrophoresis results indicated that only primers with single target bands (no miscellaneous band) were considered to be primers that met the needs (as shown in Supplementary Materials Figure S1). PCR primer sequences are shown in Table 2. Each qRT-PCR was performed with three independent biological samples and was in triplicate. The results obtained were normalized to the expression level of the housekeeping gene β-actin, and relative mRNA expression levels were expressed as 2^−^^△△CT^. Calculations were performed using Microsoft Excel.

### 5.5. Statistics

The student’s test was used to test for statistical differences across groups. The statistical significance was set at *p* ≤0.05. * indicates *p* ≤0.05, ** indicates *p* ≤0.01 and *** indicates *p* <0.001. Data are expressed as the mean ± standard error.

## Figures and Tables

**Table 1:** Physical index.

Groups	Weight Gains (g)	Intestinal Length / mice heights (cm)	Water Consumption (mL)
Control (0 μg/L)	7.24 ± 0.89	4.34 ± 0.22	4074.5 ± 126.50
1 μg/L	7.66 ± 0.51	4.23 ± 0.16	3651.5 ± 202.07
30 μg/L	6.32 ± 0.48	4.05 ± 0.20	4178 ± 99.70
60 μg/L	6.65 ± 0.43	4.30 ± 0.22	3732 ± 166.45
90 μg/L	5.91 ± 0.70	4.20 ± 0.15	4110 ± 107.38
120 μg/L	6.03 ± 0.69	4.33 ± 0.17	4008 ± 77.92

**Table 2:** Specific primers used for RT-PCR in this study.

Primer	Sequence (5’→3’)	Annealing temperature (°C)	Product length (bp)
TNF-α	Forward: GTGCCTATGTCTCAGCCTCT Reverse: AGGCTTGTCACTCGAATTTTGA	60	187
IL-8	Forward: TGGGTGAAGGCTACTGTTGG Reverse: AGGCTTTTCATGCTCAACACT	60	114
IL-10	Forward: ATAACTGCACCCACTTCCCA Reverse: GGGCATCACTTCTACCAGGT	60	206
TGF-β1	Forward: AGGGCTACCATGCCAACTTC Reverse: CCACGTAGTAGACGATGGC	60	168
IL-1β	Forward: AAGGGCTGCTTCCAAACCTTTGAC Reverse: ATACTGCCTGCCTGAAGCTCTTGT	60	100
β-actin	Forward: CTAAGGCCAACCGTGAAAAG Reverse: ACCAGAGGCATACAGGGACA	60	104

## Supplementary Materials

The following are available online at https://www.mdpi.com/2072-6651/11/9/482/s1, Figure S1: Suitability detection of primers, Figure S2: Quantification of goblet cells in Figure 1B.

## References

[1] Carmichael W.W., Azevedo S.M.F.O., An J.S., Molica R.J.R., Jochimsen E.M., Lau S., Rinehart K.L., Shaw G.R., Eaglesham G.K. (2001). Human Fatalities from Cyanobacteria: Chemical and Biological Evidence for Cyanotoxins. Environ. Health. Perspect..

[2] Chia M.A., Kramer B.J., Jankowiak J.G., Bittencourt-Oliveira M.D.C., Gobler C.J. (2019). The Individual and Combined Effects of the Cyanotoxins, Anatoxin-a and Microcystin-LR, on the Growth, Toxin Production, and Nitrogen Fixation of Prokaryotic and Eukaryotic Algae. Toxins.

[3] Yang F., Wen C., Zheng S., Yang S., Chen J., Feng X. (2018). Involvement of MAPK/ERK1/2 pathway in microcystin-induced microfilament reorganization in HL7702 hepatocytes. J. Toxicol. Environ. Health Part A.

[4] Yang F., Guo J., Huang F.Y., Massey I.Y., Huang R.X., Li Y.H., Wen C., Ding P., Zeng W.M., Liang G.Y. (2018). Removal of Microcystin-LR by a Novel Native Effective Bacterial Community Designated as YFMCD4 Isolated from Lake Taihu. Toxin.

[5] Lin W., Guo H., Wang L., Zhang D., Wu X., Li L., Li D., Tang R. (2018). Nitrite Enhances MC-LR-Induced Changes on Splenic Oxidation Resistance and Innate Immunity in Male Zebrafish. Toxins.

[6] Zhang Y., Zhuang H., Yang H., Xue W., Wang L., Wei W. (2019). Microcystin-LR disturbs testicular development of giant freshwater prawn Macrobrachium rosenbergii. Chemosphere.

[7] Spoof L., Catherine A., Meriluto J., Spoof L., Codd G.A. (2017). Appendix 3. Handbook of Cyanobacterial Monitoring and Cyanotoxin Analysis.

[8] Chen J., Xie P. (2005). Tissue distributions and seasonal dynamics of the hepatotoxic microcystins-LR and -RR in two freshwater shrimps, Palaemon modestus and Macrobrachium nipponensis, from a large shallow, eutrophic lake of the subtropical China. Toxicon.

[9] Massey I.Y., Yang F., Ding Z., Yang S., Guo J., Tezi C., Al-Osman M., Kamegni R.B., Zeng W. (2018). Exposure routes and health effects of microcystins on animals and humans: A mini-review. Toxicon.

[10] Papadimitriou T., Kagalou I., Stalikas C., Pilidis G., Leonardos I.D. (2012). Assessment of microcystin distribution and biomagnification in tissues of aquatic food web compartments from a shallow lake and evaluation of potential risks to public health. Ecotoxicology.

[11] Wu J.-X., Huang H., Yang L., Zhang X.-F., Zhang S.-S., Liu H.-H., Wang Y.-Q., Yuan L., Cheng X.-M., Zhuang D.-G. (2018). Gastrointestinal toxicity induced by microcystins. World J. Clin. Cases.

[12] Chen L., Yang S., Wen C., Zheng S., Yang Y., Feng X., Chen J., Luo D., Liu R., Yang F. (2019). Regulation of Microcystin-LR-Induced DNA Damage by miR-451a in HL7702 Cells. Toxins.

[13] Mrdjen I., Morse M.A., Ruch R.J., Knobloch T.J., Choudhary S., Weghorst C.M., Lee J. (2018). Impact of Microcystin-LR on Liver Function Varies by Dose and Sex in Mice. Toxins.

[14] Liu H., Zhang S., Liu C., Wu J., Wang Y., Yuan L., Du X., Wang R., Marwa P.W., Zhuang D. (2018). Resveratrol Ameliorates Microcystin-LR-Induced Testis Germ Cell Apoptosis in Rats via SIRT1 Signaling Pathway Activation. Toxins.

[15] Vidal F., Sedan D., D’Agostino D., Cavalieri M.L., Mullen E., Varela M.M.P., Flores C., Caixach J., Andrinolo D. (2017). Recreational Exposure during Algal Bloom in Carrasco Beach, Uruguay: A Liver Failure Case Report. Toxins.

[16] Azevedo S.M., Carmichael W.W., Jochimsen E.M., Rinehart K.L., Lau S., Shaw G.R., Eaglesham G.K. (2002). Human intoxication by microcystins during renal dialysis treatment in Caruaru—Brazil. Toxicology.

[17] WHO (1998). Cyanobacterial Toxins: Microcystin-LR. Guidelines for Drinking Water Quality.

[18] Thomson A.B., Drozdowski L., Iordache C., Thomson B.K., Vermeire S., Clandinin M.T., Wild G. (2003). Small bowel review: Normal physiology, part 1. Dig. Dis. Sci..

[19] Middendorp S., Schneeberger K., Wiegerinck C.L., Mokrý M., Akkerman R.D.L., Van Wijngaarden S., Clevers H., Nieuwenhuis E.E.S. (2014). Adult Stem Cells in the Small Intestine Are Intrinsically Programmed with Their Location-Specific Function. Stem Cells.

[20] McKie A.T., Barrow D., Latunde-Dada G.O., Rolfs A., Sager G., Mudaly E., Mudaly M., Richardson C., Barlow D., Bomford A. (2001). An Iron-Regulated Ferric Reductase Associated with the Absorption of Dietary Iron. Science.

[21] Shneider B.L. (2001). Intestinal Bile Acid Transport: Biology, Physiology, and Pathophysiology. J. Pediatr. Gastroenterol. Nutr..

[22] Kogut M.H., Genovese K.J., Swaggerty C.L., He H., Broom L. (2018). Inflammatory phenotypes in the intestine of poultry: Not all inflammation is created equal. Poult. Sci..

[23] Ungaro R., Mehandru S., Allen P.B., Peyrin-Biroulet L., Colombel J.F. (2017). Ulcerative colitis. Lancet.

[24] Rigby R.J., Hunt M.R., Scull B.P., Simmons J.G., Speck K.E., Helmrath M.A., Lund P.K. (2009). A new animal model of post-surgical bowel inflammation and fibrosis: The effect of commensal microflora. Gut.

[25] Medzhitov R. (2008). Origin and physiological roles of inflammation. Nature.

[26] Secombes C., Wang T., Hong S., Peddie S., Crampe M., Laing K., Cunningham C., Zou J., Laing K. (2001). Cytokines and innate immunity of fish. Dev. Comp. Immunol..

[27] Tran H.B., Chen S.-C., Chaung H.-C., Cheng T.-C. (2019). Molecular cloning of IL-6, IL-10, IL-11, IFN-ɤ and modulation of pro- and anti-inflammatory cytokines in cobia (Rachycentron canadum) after Photobacterium damselae subsp. piscicida infection. Comp. Biochem. Physiol. Part B Biochem. Mol. Boil..

[28] Weel J.F., Hopman C.T., Van Putten J.P. (1989). Stable expression of lipooligosaccharide antigens during attachment, internalization, and intracellular processing of Neisseria gonorrhoeae in infected epithelial cells. Infect. Immun..

[29] Brundin L., Bryleva E.Y., Thirtamara Rajamani K. (2017). Role of Inflammation in Suicide: From Mechanisms to Treatment. Neuropsychopharmacology.

[30] Yoshie O., Imai T., Nomiyama H. (2001). Chemokines in immunity. Adv. Immunol..

[31] Zuena A.R., Casolini P., Lattanzi R., Maftei D. (2019). Chemokines in Alzheimer’s Disease: New Insights into Prokineticins, Chemokine-Like Proteins. Front. Pharmacol..

[32] Liu N., Chen R., Du H., Wang J., Zhang Y., Wen J. (2009). Expression of IL-10 and TNF-alpha in rats with cerebral infarction after transplantation with mesenchymal stem cells. Cell. Mol. Immunol..

[33] Kim S.K., Barron L., Hinck C.S., Petrunak E.M., Cano K.E., Thangirala A., Iskra B., Brothers M., Vonberg M., Leal B. (2017). An engineered transforming growth factor beta (TGF-beta) monomer that functions as a dominant negative to block TGF-beta signaling. J. Biol. Chem..

[34] Alijotas-Reig J., Esteve-Valverde E., Ferrer-Oliveras R., Llurba E., Gris J.M. (2017). Tumor Necrosis Factor-Alpha and Pregnancy: Focus on Biologics. An Updated and Comprehensive Review. Clin. Rev. Allergy Immunol..

[35] Loboda A., Sobczak M., Jozkowicz A., Dulak J. (2016). TGF-β1/Smads and miR-21 in Renal Fibrosis and Inflammation. Mediat. Inflamm..

[36] Strober W., Fuss I.J., Blumberg R.S. (2002). The immunology of mucosal models of inflammation. Annu. Rev. Immunol..

[37] Mehaffey E., Majid D.S.A. (2017). Tumor necrosis factor-α, kidney function, and hypertension. Am. J. Physiol. Physiol..

[38] Chen J., Xie P., Li L., Xu J. (2009). First Identification of the Hepatotoxic Microcystins in the Serum of a Chronically Exposed Human Population Together with Indication of Hepatocellular Damage. Toxicol. Sci..

[39] Li Y., Chen J.A., Zhao Q., Pu C., Qiu Z., Zhang R., Shu W. (2011). А сrоѕѕ-ѕесtіоnаl іnvеѕtіgаtіоn оf сhrоnіс ехр оѕurе tо mісrосуѕtіn іn relationship to childhood liver damage in the Three Gorges R eservoir Region, China. Environ. Health Perspect..

[40] Sedan D., Laguens M., Copparoni G., Aranda J.O., Giannuzzi L., Marra C.A., Andrinolo D. (2015). Hepatic and intestine alterations in mice after prolonged exposure to low oral doses of Microcystin-LR. Toxicon.

[41] Su R.C., Blomquist T.M., Kleinhenz A.L., Khalaf F.K., Dube P., Lad A., Breidenbach J.D., Mohammed C.J., Zhang S.G., Baum C.E. (2019). Exposure to the Harmful Algal Bloom (HAB) Toxin Microcystin-LR (MC-LR) Prolongs and Increases Severity of Dextran Sulfate Sodium (DSS)-Induced Colitis. Toxins.

[42] Pan C., Chen Y., Xu T., Wang J., Li D., Han X. (2018). Chronic exposure to microcystin-leucine-arginine promoted proliferation of prostate epithelial cells resulting in benign prostatic hyperplasia. Environ. Pollut..

[43] Baumgart D.C., Dignass A.U. (2002). Intestinal barrier function. Curr. Opin. Clin. Nutr..

[44] Ren Z., Guo C., Yu S., Zhu L., Wang Y., Hu H., Deng J. (2019). Progress in Mycotoxins Affecting Intestinal Mucosal Barrier Function. Int. J. Mol. Sci..

[45] Peterson L.W., Artis D. (2014). Intestinal epithelial cells: Regulators of barrier function and immune homeostasis. Nat. Rev. Immunol..

[46] Ito E., Kondo F., Terao K., Harada K.-I. (1997). Neoplastic nodular formation in mouse liver induced by repeated intraperitoneal injections of microcystin-LR. Toxicon.

[47] Ito E., Kondo F., Harada K.-I. (2000). First report on the distribution of orally administered microcystin-LR in mouse tissue using an immunostaining method. Toxicon.

[48] Chen C., Liu W., Wang L., Li J., Chen Y., Jin J., Kawan A., Zhang X. (2016). Pathological damage and immunomodulatory effects of zebrafish exposed to microcystin-LR. Toxicon.

[49] Li Y., Sun B., Wu H., Nie P. (2009). Effects of pure microcystin-LR on the transcription of immune related genes and heat shock proteins in larval stage of zebrafish (Danio rerio). Aquaculture.

[50] Rymuszka A., Sierosławska A., Bownik A., Skowroński T. (2010). Microcystin-LR modulates selected immune parameters and induces necrosis/apoptosis of carp leucocytes. Environ. Toxicol. Chem..

[51] Xia H., Song T., Wang L., Jiang L., Zhou Q., Wang W., Liu L., Yang P., Zhang X. (2018). Effects of dietary toxic cyanobacteria and ammonia exposure on immune function of blunt snout bream (Megalabrama amblycephala). Fish Shellfish. Immunol..

[52] Fenton M.J., Vermeulen M.W., Clark B.D., Webb A.C., Auron P.E. (1988). Human pro-IL-1 beta gene expression in monocytic cells is regulated by two distinct pathways. J. Immunol..

[53] Jarrous N., Kaempfer R. (1994). Induction of human interleukin-1 gene expression by retinoic acid and its regulation at processing of precursor transcripts. J. Boil. Chem..

[54] Zheng J., Wang J., Pouliot M., Authier S., Zhou D., Loose D.S., Hauer-Jensen M. (2015). Gene expression profiling in non-human primate jejunum, ileum and colon after total-body irradiation: A comparative study of segment-specific molecular and cellular responses. BMC Genom..

[55] Zimmermann H.W., Seidler S., Gassler N., Nattermann J., Luedde T., Trautwein C., Tacke F. (2011). Interleukin-8 Is Activated in Patients with Chronic Liver Diseases and Associated with Hepatic Macrophage Accumulation in Human Liver Fibrosis. PLoS ONE.

[56] Freire-De-Lima C.G., Xiao Y.Q., Gardai S.J., Bratton D.L., Schiemann W.P., Henson P.M. (2006). Apoptotic Cells, through Transforming Growth Factor-β, Coordinately Induce Anti-inflammatory and Suppress Pro-inflammatory Eicosanoid and NO Synthesis in Murine Macrophages. J. Boil. Chem..

[57] Savill J., Fadok V. (2000). Corpse clearance defines the meaning of cell death. Nature.

[58] Voll R.E., Herrmann M., Roth E.A., Stach C., Kalden J.R., Girkontaite I. (1997). Immunosuppressive effects of apoptotic cells. Nature.

[59] Savill J., Dransfield I., Gregory C., Haslett C. (2002). A blast from the past: Clearance of apoptotic cells regulates immune responses. Nat. Rev. Immunol..

[60] Shen Y., Zhang C., Chen Y., Chen Y. (2015). TGF-β in Inflammatory Bowel Diseases: A Tale of the Janus-Like Cytokine. Crit. Rev. Eukaryot. Gene Expr..

[61] Ruemmele F.M., Garnier-Lengliné H. (2013). Transforming Growth Factor and Intestinal Inflammation: The Role of Nutrition. Nestle Nutr. Inst. Workshop Ser..

[62] Huang X.R., Chung A.C., Zhou K.L., Wang X.J., Lan H.Y. (2008). Latent TGF- 𝛽1 protects against crescentic glomerulonephritis. J. Am. Soc. Nephrol..

[63] Dai C., Wen X., He W., Liu Y. (2011). Inhibition of proinflammatory RANTES expression by TGF-𝛽1 is mediated by glycogen synthase kinase-3𝛽-dependent 𝛽-catenin signaling. J. Biol. Chem..

[64] Guo Y., Wang L., Ma R., Mu Q., Yu N., Zhang Y., Tang Y., Li Y., Jiang G., Zhao D. (2016). JiangTang XiaoKe granule attenuates cathepsin K expression and improves IGF-1 expression in the bone of high fat diet induced KK-Ay diabetic mice. Life Sci..

[65] Song Y., Chang H., Ji W., Peng H., Shi F., Bai Y.B., Yin J.S. (2018). Comparative study on different methods of detecting eosinophils in nasal polyps. Lin Chung Er Bi Yan Hou Tou Jing Wai Ke Za Zhi.

[66] Wen C., Yang S., Zheng S., Feng X., Chen J., Yang F. (2018). Analysis of long non-coding RNA profiled following MC-LR-induced hepatotoxicity using high-throughput sequencing. J. Toxicol. Env. Heal. A..

