# Supplementary material for: Effects of Microcystin-LR on the Microstructure and Inflammation-Related Factors of Jejunum in Mice
Source: Toxins (Basel). 2019 Aug 21;11(9):482. doi: 10.3390/toxins11090482 (PMC6783826; doi:10.3390/toxins11090482)
Supplement: Supplementary file 1 [file toxins-11-00482-s001.pdf]

Supplementary Materials: Effects of Microcystin-Leucine Arginine on the Microstructure and Inflammation-Related Factors of Jejunum in Mice

Linghui Cao, Feiyu Huang, Isaac Yaw Massey, Cong Wen, Shuilin Zheng, Shuaishuai Xu and Fei Yang *

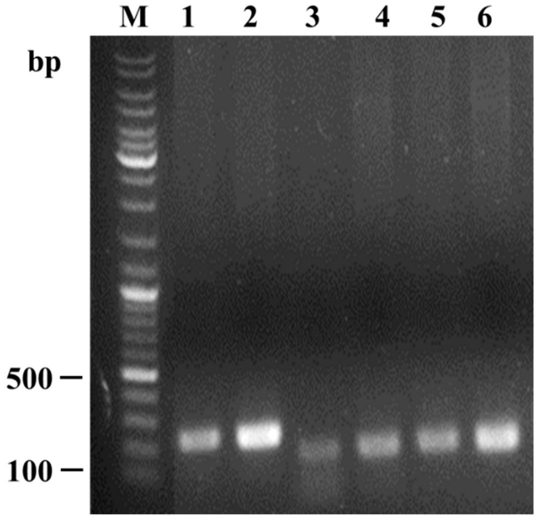

Figure 1. Suitability detection of primers. M: DNA marker, No.1, 2, 3, 4, 5 and 6 track represents PCR amplification products with TNF-α Forward / TNF-α Reverse, IL-10 Forward / IL-10 Reverse, IL-1β Forward / IL-1β Reverse, β-actin Forward / β-actin Reverse, IL-8 Forward / IL-8 Reverse, TGF-β1 Forward / TGF-β1 Reverse primer pairs respectively. There was no miscellaneous band below the target band of each primer.

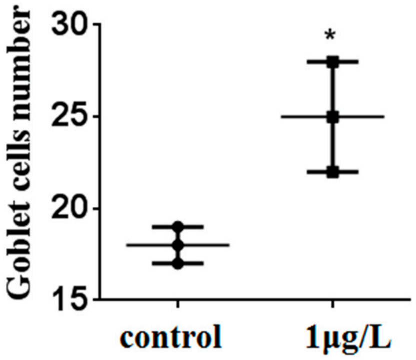

Figure S2. Quantification of goblet cells in Figure 1B. * indicates $p \leq 0.05$.
